# Calves as Main Reservoir of Antibiotic Resistance Genes in Dairy Farms

**DOI:** 10.3389/fpubh.2022.918658

**Published:** 2022-06-20

**Authors:** Barbara Salerno, Matteo Cornaggia, Raffaella Sabatino, Andrea Di Cesare, Maddalena Furlan, Lisa Barco, Massimiliano Orsini, Benedetta Cordioli, Claudio Mantovani, Luca Bano, Carmen Losasso

**Affiliations:** ^1^Laboratory of Microbial Ecology and Genomics, Istituto Zooprofilattico Sperimentale delle Venezie, Legnaro, Italy; ^2^Laboratory of Clinical Diagnostics, Istituto Zooprofilattico Sperimentale delle Venezie, Legnaro, Italy; ^3^National Research Council of Italy-Water Research Institute (CNR-IRSA), Verbania, Italy; ^4^Laboratory of Science Communication, Istituto Zooprofilattico Sperimentale delle Venezie, Legnaro, Italy

**Keywords:** antimicrobial resistance, dairy cows, antibiotics, ddPCR, spread

## Abstract

A side effect of antibiotic usage is the emergence and dissemination of antibiotic resistance genes (ARGs) within microbial communities. The spread of ARGs among pathogens has emerged as a public health concern. While the distribution of ARGs is documented on a global level, their routes of transmission have not been clarified yet; for example, it is not clear whether and to what extent the emergence of ARGs originates in farms, following the selective pressure exerted by antibiotic usage in animal husbandry, and if they can spread into the environment. Here we address this cutting edge issue by combining data regarding antimicrobial usage and quantitative data from selected ARGs (*bla*_TEM_, *bla*_CTXM_*, erm*B, *van*A, *qnr*S, *tet*A, *sul*2, and *mcr*-1) encoding for resistance to penicillins, macrolides-lincosamides-streptogramins, glycopeptides, quinolones, tetracyclines, sulfonamides, and colistin at the farm level. Results suggest that dairy farms could be considered a hotspot of ARGs, comprising those classified as the highest risk for human health and that a correlation existed between the usage of penicillins and *bla*_TEM_ abundances, meaning that, although the antibiotic administration is not exclusive, it remains a certain cause of the ARGs' selection and spread in farms. Furthermore, this study identified the role of calves as the main source of ARGs spread in dairy farms, claiming the need for targeted actions in this productive category to decrease the load of ARGs along the production chain.

## Introduction

Antibiotic resistance (AR) is one of the most impactful phenomena on the effectiveness of healthcare. In this regard, the European Center for Disease Prevention and Control (ECDC) estimates that more than 670,000 infections are caused by antibiotic-resistant bacteria each year, with approximately 33,000 people dying as a direct consequence (1), at a yearly cost of €1.5 billion (2). Furthermore, it has been estimated that multidrug-resistant (MDR) bacteria cause at least 2 million infections and 23,000 deaths per year in the United States, and a yearly cost of $55–70 billion (3). Some authors estimate that AR will cause 10 million deaths per year by 2050 (4). Infections caused by pathogenic bacteria resistant to “critically important antimicrobials” for human medicine (CIAs), such as extended-spectrum beta-lactamase (ESBLs) producers resistant to third and fourth generation cephalosporins, methicillin-resistant staphylococci, fluoroquinolone-resistant Enterobacteriaceae, and carriers of *mcr* 1-8 plasmid genes resistant to polymyxins (5), are among those considered more dangerous for human health. Antibiotics are essential for the treatment of bacterial infections in humans and animals; it is, therefore, a top priority to preserve their effectiveness. Addressing the rising threat of AR requires a holistic and multisectoral approach, referred to as the “*One Health*” approach, because antimicrobials used to treat infectious diseases in humans may be the same or similar to those used for animals. Resistant bacteria arising in animals may spread to the environment and eventually to humans, as AR does not recognize geographic nor animal-environment-human borders. Microbial communities from humans, animals, and the environment dynamically interact in bidirectional ways. This interaction includes the possibility of exchanging genes, between the relative microbiomes, with the chance to transfer new phenotypic skills, including those related to AR. The establishment of antibiotic resistance genes (ARGs) can arise either from new mutations in the bacterial genome or through the acquisition of genes encoding for resistance. The consequences of this transfer are even more alarming since this mechanism can often promote the simultaneous spread of AR to several unrelated classes of antibiotics, especially if genes for such resistance are co-located on the same mobile genetic elements (6). Once resistance has developed, bacteria may retain it for longer periods even if not exposed to antibiotics, triggering the persistence of the phenomenon over time (7). Therefore, the AR in animals and environmental microbial communities can be considered as both a direct and indirect hazard to human health. In the first case, it refers to the fact that pathogenic antibiotic-resistant bacteria can be transferred to the human microbiota and colonize it, causing antibiotic-resistant infections. In the second case, it refers to the fact that antibiotic-resistant bacteria can transfer their ARGs to pathogenic bacteria, either directly or through other, more competent, commensal bacteria. In the last case, the biological hazard is represented by the ARGs themselves. Although antibiotic-resistant microorganisms currently do not represent a burning issue in dairy farming, optimization of antimicrobial usage is one of the objectives of this industry as animal-friendly, economical, and resource-saving milk production provides the basis for sustained consumer acceptance. Antibiotics are predominantly administered for the control of udder infections (8). Bovine mastitis is a painful disease for dairy cows, representing the most economically important contagious disease on dairy farms (9, 10). Thus, antimicrobial treatment favors keeping bovine udder health, animal welfare, and economic aspects in balance.

Despite the distribution, at the global level, of genes underlying AR being well documented (11), even in dairy farms (12, 13), there are still gaps of knowledge about their origin and acquisition. In particular, it has not been clarified yet whether and to what extent the emergence of ARGs originates in dairy farms following the selective pressure exerted by antibiotic usage. The main objective of the present work is to bridge this knowledge gap by investigating the relationships between antibiotic usage and the selection of ARGs in the context of bovine milk production. Moreover, the spread of ARGs within the farm was quantitatively assessed, comparing their load in different stages of the farming process.

## Materials and Methods

### Sampling Scheme

In total, 10 dairy farms located in four different provinces (A, B, C, D, E, F, G, H, I, and J) distributed in Northeastern Italy were visited in 2020. Each farm housed between 40 and 250 cows. The farms were loose housing systems consisting of cubicle houses with slatted floors.

The sampling size was based on the number of bovines in the herd. In herds with more than 150 bovines, individual fecal samples were collected from the rectum of 20 lactating cows, 10 dry cows, 10 heifers (female heifers aged from 2 to 24 months), and 5 calves (animals <2 months old). In herds with <150 bovines, individual fecal samples were collected from the rectum of 15 lactating cows, 5 dry cows, 5 heifers, and 5 calves. Farms C, G, and H were considered small (<150 animals total), while A, B, D, E, F, I, and J were considered as big farms (>150 animals total). Samples were immediately transported to the laboratory under refrigeration conditions.

### Antibiotic Consumption

Data on antimicrobial consumption were accessible *via* the information system of the Italian Integrated Program for the Classification of Intensive Animal Farming (*ClassyFarm*) provided by the General Directorate of Animal Health and Veterinary Medicines of the Ministry of Health (https://www.classyfarm.it/). These data are calculated on the basis of Electronic Veterinary Prescription (REV) and expressed in Defined Daily Doses Animal for Italy (DDDAit). ClassyFarm provides data about both the general consumption of antimicrobials (DDDAit/farm) and the specific antimicrobial consumption divided for each antibiotic class, i.e., penicillins, macrolides-lincosamides-streptogramins (MLS), tetracyclines, and sulfonamides.

### Antibiotic Resistance Determinants Selection

The following resistance genetic determinants were tested:

*bla*_TEM_ and *bla*_CTXM_ are representative genes encoding for resistance to b-lactams; *qnr*S is a quinolone resistance gene; *sul*2 and *tet*A are resistance genes against two of the oldest discovered antibiotics, sulfonamides and tetracycline, respectively; *erm*B is a representative of genes encoding for MLS resistance; and *van*A is a representative resistance gene for glycopeptides. Furthermore, *mcr*-1 was selected as a particularly relevant ARG at the clinical level. The eight selected ARGs were first screened by end-point PCR and, where positive, were quantified by digital droplet PCR (ddPCR).

### Sample Processing and DNA Extraction

Fecal samples were pooled together in groups of five. Each final sample was stomached for 1 min at room temperature to obtain a homogenized sample. A volume of 0.2 ml was used for DNA extraction. Three replicates were processed for each sample.

DNA extraction was performed as follows: samples were placed in lysis buffer (500 mM NaCl, 50 mM Tris-HCl pH 8.0, 50 mM EDTA, and 4% sodium dodecyl sulfate), for 20 min at 70°C. After centrifugation at 5,000 × g for 5 min, the supernatant was transferred to a tube containing 200 μl of 10 mM ammonium acetate. Tubes were incubated on ice for 5 min and then centrifuged at 5,000 × g for 5 min. The supernatant was transferred and incubated with an equal volume of 99.8% isopropanol for 30 min on ice and then centrifuged at 16,000 × g for 15 min. The DNA pellet was washed with 70% ethanol and resuspended with 10 mM Tris EDTA. The DNA pellet was treated with Proteinase K and incubated in Buffer AL of the Qiagen QIAamp DNA Mini Kit according to kit instructions. The DNA concentration was measured with the NanoDrop One Microvolume UV-Vis Spectrophotometer (ThermoFisher).

### Genes Detection and Quantification

The DNA samples were analyzed by end-point PCR (testing all the above-mentioned ARGs) and ddPCR. End-point PCR assays were carried out in 25 μl with 2.5 μl of DNA (with a range between 4.75 and 69 ng), 0.4 μM of each primer, 2 mM of MgCl2, 200 μM of dNTPs, 1X PCR Buffer II (Thermo Fisher Scientific), and 2.5 U of AmpliTaq Gold DNA Polymerase (Thermo Fisher Scientific). The PCR program was 95°C for 2 min, 25 cycles of 95°C for 30 s, annealing temperature for 30 s, 72°C for 30 s, and the final extension was set at 72°C for 5 min (Supplementary Table 1). PCR products were run in agarose electrophoresis gel at 2%. Only positive samples by PCR were tested with ddPCR. DNA extracts from *S. Typhimurium* 2011_2776 for *bla*_TEM_, *sul*2, and *tet*A, *Escherichia coli* 2019_82 for *mcr*-1, monophasic variant of *Salmonella enterica* serovar Typhimurium 2019_112 for *qnr*S, *Campylobacter jejuni* for *erm*B, and *Enterococcus faecium* for *van*A and *bla*_CTXM_ were used as positive controls. Positive DNA samples by end-point PCR were 10-fold-diluted before the analysis with ddPCR. ddPCR assays were carried out with 22 μl of reaction mix prepared by assembling the QX200 ddPCR EvaGreen Supermix with primers at the concentration of 3 μM and 2 μl of DNA and nuclease-free water. Aliquots of 20 μl of each sample were transferred to the DG8 Cartridge together with 70 μl of QX200 Droplet Generation Oil. The DG8 Cartridge was placed in the QX200 Droplet Generator (Bio-Rad). Droplets were carefully transferred to a 96-well PCR plate for the amplification on a C1000 Touch Thermal Cycler (Bio-Rad). Positive controls (amplified target gene) and no template controls (NTC) were included in each run. The program, recommended by Bio-Rad, was 95°C for 5 min, 40 cycles of 95°C for 30 s, and annealing/extension temperature (optimized for each tested gene) for 1 min with a ramp rate of 2°C s^−1^ and two final steps at 4°C for 5 min and 90°C for 5 min (Supplementary Table 1). The plates were transferred to a QX200 Droplet Reader (Bio-Rad) to acquire data. Reactions with more than 10,000 droplets were analyzed. Thresholds to discriminate between positive and negative droplets were manually set up and only samples with ≥3 positive droplets (14) were considered as positive. Data were expressed as gene copy μl^−1^ using QuantaSoft Analysis Pro software (Bio-Rad) for the analysis. The ARG abundances were normalized by dividing their copy number per copy of the 16S rRNA gene.

### Statistical Analyses

To evaluate the dynamics of ARGs in our system, the difference in abundance of *bla*_TEM_, *erm*B, *sul*2, and *tet*A genes according to animal category (four levels: calves, heifers, lactating cows, and dry cows) and farm (ten levels: A, B, C, D, E, F, G, H, I, and J) was assessed, first, by MANOVA, analyzing genes collectively, and, then, by ANOVA, with Tukey's *post-hoc* test, for each single gene. In addition, the differences in the total abundance of ARGs were evaluated by ANOVA (Tukey's *post-hoc* test) as well. The analyses were conducted in the R environment v3.6 (15).

The correlation between the relative total abundances of ARGs and total consumption of antibiotics in each farm was determined using Pearson's correlation, considering them as correlated for r ≥ |0.75|. Moreover, the correlation between the relative abundance of *bla*_TEM_, *erm*B, *sul*2, and *tet*A and the consumption of the corresponding antibiotic (i.e., penicillins, MLS, sulfonamides, and tetracyclines, respectively) was evaluated *via* Pearson's analysis too.

## Results

### Antibiotic Consumption

Annual antimicrobial consumption data were extrapolated directly from *ClassyFarm* through the univocal farm code provided by the Italian National Institute of Statistics (ISTAT). Results expressed in DDDAit are reported in Table 1. An incisive data analysis was conducted to extrapolate antimicrobial consumption data regarding the pharmaceutical categories related to the targeted genes (i.e., penicillins, MLS, sulfonamides, and tetracyclines).

**Table 1:** Annual antimicrobial consumption referred to 2019 of enrolled farms.

**Farm**	**Penicillins**	MLS*	**Sulfonamides**	**Tetracyclines**	**Total**
A	0.000	0.000	0.000	0.000	0.000
*B*	0.035	0.000	0.038	0.452	1.180
*C*	0.381	0.076	0.680	0.219	1.680
*D*	0.102	0.067	0.000	0.030	1.620
*E*	4.543	0.000	0.038	0.000	5.100
*F*	0.000	0.000	0.000	0.000	0.000
*G*	0.000	0.000	0.000	0.000	0.000
*H*	1.304	0.334	0.111	0.214	3.150
*I*	0.000	0.000	0.000	0.000	0.000
*J*	3.628	0.090	0.000	0.000	5.130

Data are expressed in Defined Daily Doses Animal for Italy (DDDAit) and extrapolated directly from the Classification of Intensive Animal Farming (ClassyFarm) information system based on the veterinary electronic prescription. Antimicrobials were categorized according to the World Health Organization (WHO). The total amount of antimicrobials includes all the active prescribed molecules used in the stable. *MLS, macrolides-lincosamides-streptogramins.

### Antibiotic Resistance Genes' Presence and Abundance

The presence of eight ARGs (*bla*_CTXM_, *bla*_TEM_, *erm*B, *qnr*S, *sul*2, *tet*A, *van*A, and *mcr*-1) was investigated in different animal categories by PCR. Among these, *bla*_TEM_, *erm*B, *sul*2, and *tet*A were positive in at least one of the evaluated samples; whereas *bla*_CTXM_, *qnr*S, *van*A, and *mcr*-1 were never detected (Supplementary Table 2). In detail, *bla*_TEM_ and *erm*B were detected in calves of several farms (D, E, and G for the former; A, D, F, G, H, I, and J for the latter); *tet*A was present both in calves of different farms (A, D, E, F, and G) and in lactating cows from farm D; *sul*2 was found in the fecal samples of calves (farms D, F, G, H, I, and J), lactating cows (farms F and G), and dry cows (farms F and G) (Supplementary Table 2). All the samples from farms B and C were negative for the tested genes (Supplementary Table 2). Positive genes were then quantified by ddPCR. When quantifiable, their abundance ranged from 6.5 × 10^−6^ to 2.96 × 10^−1^ gene copies/16S rRNA gene copy (Supplementary Table 3). Specifically, *bla*_TEM_ and *erm*B were always quantifiable and their concentration was comprised of between 3.62 × 10^−5^ and 5.37 × 10^−2^ gene copies/16S rRNA gene copy for the former, and between 2.94 × 10^−5^ and 2.96 × 10^−1^ gene copies/16S rRNA gene copy for the latter (Supplementary Table 3). In addition, *sul*2 ranged from 6.5 × 10^−6^ to 1.82 × 10^−1^ gene copies/16S rRNA gene copy; whereas *tet*A from 9.4 × 10^−6^ to 3.89 × 10^−2^ gene copies/16S rRNA gene copy (Supplementary Table 3).

**Figure 1:**
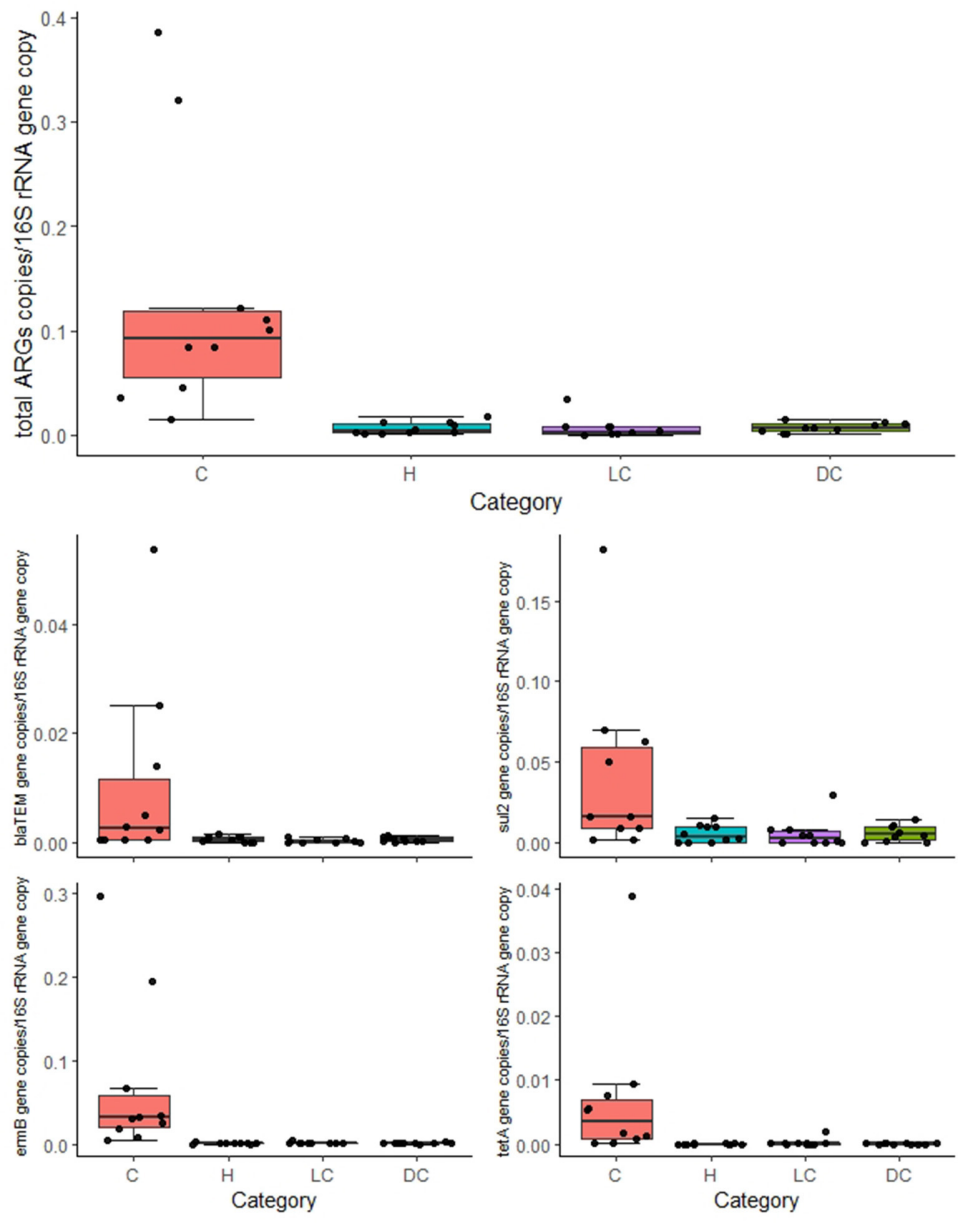
Normalized abundances of genes according to animal category. Boxplots of the distribution of abundances of total antibiotic resistance genes (ARGs), *bla*_TEM_, *erm*B, *sul*2, and *tet*A within bacterial communities in calves (C), heifers (H), lactating cows (LC), and dry cows (DC). The thick horizontal line represents the median, the box represents 50% of the values, the whiskers extend to the highest and lowest value within the 1.5 interquartile range (IQR), and the dots represent the single observations.

The overall normalized abundance of tested genes changed significantly according to the animal category (MANOVA: Pillai's trace = 0. 8754, *F* = 2.6782, and *p* = 0.004572) (Supplementary Table 4). In all the cases, the genes were more abundant in calves with respect to heifers, lactating cows, and dry cows (ANOVA: *p* ≤ 0.00228) (Figure 1; Table 2). The same was observed by analyzing the total ARG abundances (ANOVA: *F* = 22.061, *p* = 1.99e-07) (Figure 1). On the contrary, when considering the farm as a factor, no significant differences were seen among the samples (*p* ≥ 0.3867) (Table 2).

**Table 2:** Statistical results for the analysis of variance (ANOVA) assessing the influence of the experimental variables (animal category and farm) on the normalized abundance of total antibiotic resistance genes (ARGs), *bla*_TEM_, *erm*B, *sul*2, and *tet*A.

	**Df**	**Sum Sq**	**Mean Sq**	**F-value**	***p*-value**	***Post-hoc* grouping**
**Total ARGs**
Category	3	0.5250	0.17501	22.061	1.99e-07***	
*Calves*						a
*Heifers*						b
*Lactating cows*						b
*Dry cows*						b
Farm	9	0.0944	0.01049	1.322	0.272	
***bla*_TEM_**
Category	3	0.02474	0.008247	6.707	0.00158**	
*Calves*						a
*Heifers*						b
*Lactating cows*						b
*Dry cows*						b
Farm	9	0.01556	0.001729	1.406	0.23441	
***erm*** **B**
Category	3	0.31283	0.10428	15.974	3.6e-06***	
*Calves*						a
*Heifers*						b
*Lactating cows*						b
*Dry cows*						b
Farm	9	0.06791	0.00755	1.156	0.361	
***sul*** **2**
Category	3	0.09374	0.031248	6.269	0.00228**	
*Calves*						a
*MANZE*						b
*Lactating cows*						b
*Dry cows*						b
Farm	9	0.06608	0.007342	1.473	0.20801	
***tet*** **A**
Category	3	0.028609	0.009536	11.830	3.96e-05***	
*Calves*						a
*Heifers*						b
*Lactating cows*						b
*Dry cows*						b
Farm	9	0.000479	0.0000959	0.717	0.6130	

**Means p < 0.01, ***means p < 0.001.

### Relation Between Antibiotic Consumption and Antibiotic Resistance Gene Abundances

The Pearson analysis revealed no correlation between the relative total abundance of ARGs and the total consumption of antibiotics (*r* = −0.0705 and *p* = 0.8465) (Table 3). Looking at the single genes, the relative abundance of *bla*_TEM_ was significantly correlated with the consumption of penicillins (*r* = 0.8849 and *p* = 0.0007) in the farms (Table 3); whereas no correlation with the consumption of antibiotics was found for the other genes (Table 3).

**Table 3:** Pearson's correlation between ARGs and the consumption of antibiotics.

**Pairwise**		***R^**2**^***	***p*-value**
Total ARGs	- Total antibiotics	−0.07053958	0.8465
*bla*_TEM_	- Penicillins	0.8848681	0.0006675
*erm*B	- MLS*	−0.07893444	0.8284
*sul*2	- Sulfonamides	−0.3185386	0.3697
*tet*A	- Tetracyclines	−0.3662022	0.298

^*^MLS, macrolides-lincosamides-streptogramins.

## Discussion

The relative abundances of the tested ARGs were in the range of those generally found in other Italian animal farms (16, 17) and farms sampled in other countries (18, 19). This result highlights once more the need to improve knowledge about the role of the farms as hotspots of AR, threatening the farmworkers' (20) and environmental health (21) and, therefore, constituting a general concern for human health. In detail, the most abundant quantified ARG was *erm*B with a total concentration of 7.32 × 10^−1^ gene copies/16S rRNA gene copy. This gene, responsible for the resistance against MLS, has been recently classified in the rank I ARG family, thus identified as at the highest risk for human health (22). Another ARG classified in rank I (22) was *bla*_TEM_, which was the third most abundant quantified ARG in this study (total concentration of 1.22 × 10^−1^ gene copies/16S rRNA gene copy). The second most abundant ARG was *sul*2, which is widespread and constitutively present in the environment (7, 14) and, thus, it is not surprising to find a high concentration of this gene in fecal samples. Finally, although *tet*A, together with *sul*2, is commonly found in the environment (7), and tetracyclines were used in almost half of the farms immediately before the sampling, it was the ARG detected at the lowest abundance resulting in a negative result for different samples. Notably, *bla*_CTXM_, *qnr*S, and *mcr*-1, all classified as rank I ARGs (22) besides *van*A, encoding for vancomycin resistance, were not detected in any farm. This result supports the perspective of limiting the spread of such concerning genes.

Interestingly, a significant correlation between the use of β-lactams, based on prescribed penicillins, and the concentration of *bla*_TEM_ was found. A correlation between the use of antibiotics and antibiotic resistance has previously been found in humans (23–25). However, to the best of our knowledge, this kind of result was never detected in cow farms comparing the total amount of prescribed antibiotics (grouped per class) with the concentration of the selected ARGs quantified by ddPCR. This result is particularly relevant in light of the fact that several farms were sampled in this study, and each of them was sampled in different categories of the production chain. The same was not true for the other tested ARGs, meaning that factors other than antibiotic use or not drive ARG abundances in microbial communities, as extensively demonstrated previously (26–28).

As stated above, in the present study, we investigated the dynamics of ARGs along the production chain of different dairy farms, finding that, for each single tested ARG, and even for their total abundance, the calves category was richer in ARGs if compared with the other ones. This result is particularly relevant, taking into account that the main driver of the ARG abundance was the category of sampling (and not the sampled farm), suggesting that something related to the diet in the early stage of cow life influences the abundance of ARGs in their gut. This result is in agreement with what was previously found by Liu et al. (29), who attributed the role of the main source of ARGs in the calf gut to the colostrum. However, our result is not limited to the calves at different early stages of their life as done by Liu et al. (29), but extended to the comparison between the different categories of breeding, i.e., calves, heifers, lactating cows, and dry cow, meaning that ARG load decreases along the whole production chain, highlighting clearly which step of the production deserves more attention to reduce the abundance of the ARGs.

## Conclusion

This study demonstrated that dairy farms could be considered a hotspot of ARGs, comprising those classified as the highest risk for human health, and that a correlation between the prescription of penicillins and *bla*_TEM_ abundance was found, meaning that, although the antibiotic administration is not exclusive, it remains a certain cause of ARG selection and spread in farms. Furthermore, this study identified the calves category of the production chain as the main source of ARGs in dairy farms, suggesting that more actions to decrease the load of ARGs and focusing on that step to benefit whole farm activity are needed. It is, therefore, crucial to re-emphasize the role that the farm environment plays as a reservoir in maintaining ARGs and to establish surveillance systems able to figure out their load and significance.

## Data Availability Statement

The original contributions presented in the study are included in the article/Supplementary Material, further inquiries can be directed to the corresponding author/s.

## Ethics Statement

Ethical review and approval was not required for the animal study because the samples were from environmental origin.

## Author Contributions

BS and MC: investigation and data curation. RS: software and formal analysis. AD: methodology, validation, and writing—original draft. MF: investigation. LBar: conceptualization. MO: conceptualization and methodology. BC: writing—original draft. CM: conceptualization and visualization. LBan: conceptualization, resources, supervision, and funding acquisition. CL: conceptualization, methodology, validation, resources, writing—original draft, supervision, project administration, and funding acquisition. All authors contributed to the article and approved the submitted version.

## Funding

This study was funded by the Fondo Europeo Agricolo per lo Sviluppo Rurale, Programma di sviluppo rurale per il Veneto 2014-2020 (PRS), Project no. 4115471.

## Conflict of Interest

The authors declare that the research was conducted in the absence of any commercial or financial relationships that could be construed as a potential conflict of interest.

## Publisher's Note

All claims expressed in this article are solely those of the authors and do not necessarily represent those of their affiliated organizations, or those of the publisher, the editors and the reviewers. Any product that may be evaluated in this article, or claim that may be made by its manufacturer, is not guaranteed or endorsed by the publisher.
