# Supplementary material for: Calves as Main Reservoir of Antibiotic Resistance Genes in Dairy Farms
Source: Front Public Health. 2022 Jun 20;10:918658. doi: 10.3389/fpubh.2022.918658 (PMC9251204; doi:10.3389/fpubh.2022.918658)
Supplement: Supplementary file 1 [file Data_Sheet_1.docx]

**Supplementary file**

**Calves as main source for the spreading of antibiotic resistance genes to dairy farm workers and environment**

**Authors:** Barbara Salerno^*^, Matteo Cornaggia^*^, Raffaella Sabatino, Andrea di Cesare, Maddalena Furlan, Lisa Barco, Massimiliano Orsini, Benedetta Cordioli, Claudio Mantovani, Luca Bano and Carmen Losasso^#^

**Supplementary Table 1.** Primers and annealing temperature used for both end-point PCR and ddPCR.

| **Gene** | **Primer sequence (5’-3’)** | **Amplicon size (bp)** | **Annealing Temperature (°C) end-point PCR** | **Annealing Temperature (°C) ddPCR** | **Reference** |
| --- | --- | --- | --- | --- | --- |
| *bla*_TEM_ | Fw  TTCCTGTTTTTGCTCACCCAG  Rv  CTCAAGGATCTTACCGCTGTTG | 112 | 58 | 56.3 | (Di Cesare et al., 2015) |
| *bla*_CTXM_ | Fw  CTATGGCACCACCAACGATA  Rv  ACGGCTTTCTGCCTTAGGTT | 103 | 60 |  | (Di Cesare et al., 2015) |
| *tet*A | Fw  GCTACATCCTGCTTGCCTTC  Rv  CATAGATCGCCGTGAAGAGG | 210 | 64 | 59.6 | (Petrin et al., 2019) |
| *qnr*S | Fw  GACGTGCTAACTTGCGTGAT  Rv  TGGCATTGTTGGAAACTTG | 118 | 54 |  | (Petrin et al., 2019) |
| *mcr-*1 | Fw  ACACTTATGGCACGGTCTATG  Rv  GCACACCCAAACCAATGATAC | 120 | 63 | - | (Petrin et al., 2019) |
| *sul*2 | Fw TCCGGTGGAGGCCGGTATCTGG  Rv  CGGGAATGCCATCTGCCTTGAG | 191 | 60 | 58.5 | (Di Cesare et al., 2015) |
| *vanA* | Fw  GCTGTGAGGTCGGTTGTG  Rv  GCTCGACTTCCTGATGAATACG | 101 | 57 |  | (Dehbashi et al., 2020) |

REFERENCES

Dehbashi, S., Tahmasebi, H., Sedighi, P., Davarian, F., Arabestani, M.R., 2020. Development of high-resolution melting curve analysis in rapid detection of vanA gene, Enterococcus faecalis, and Enterococcus faecium from clinical isolates. Trop. Med. Health 48, 1–12. https://doi.org/10.1186/S41182-020-00197-9/FIGURES/8

Di Cesare, A., Eckert, E.M., Teruggi, A., Fontaneto, D., Bertoni, R., Callieri, C., Corno, G., 2015. Constitutive presence of antibiotic resistance genes within the bacterial community of a large subalpine lake. Mol. Ecol. 24, 3888–3900. https://doi.org/10.1111/mec.13293

Petrin, S., Patuzzi, I., Di Cesare, A., Tiengo, A., Sette, G., Biancotto, G., Corno, G., Drigo, M., Losasso, C., Cibin, V., 2019. Evaluation and quantification of antimicrobial residues and antimicrobial resistance genes in two Italian swine farms. Environ. Pollut. 255. https://doi.org/10.1016/j.envpol.2019.113183

**Supplementary Table 2.** Detection frequency of ARGs.

| Farms and categories | *bla*_CTXM_ | *bla*_TEM_ | *erm*B | *mcr*1 | *qnr*S | *sul*2 | *tet*A | *van*A |
| --- | --- | --- | --- | --- | --- | --- | --- | --- |
| A lacting cow | N | N | N | N | N | N | N | N |
| A dry cow | N | N | N | N | N | N | N | N |
| A heifers | N | N | N | N | N | N | N | N |
| A calves | N | N | P | N | N | N | P | N |
| B lacting cow | N | N | N | N | N | N | N | N |
| B dry cow | N | N | N | N | N | N | N | N |
| B heifers | N | N | N | N | N | N | N | N |
| B calves | N | N | N | N | N | N | N | N |
| C lacting cow | N | N | N | N | N | N | N | N |
| C dry cow | N | N | N | N | N | N | N | N |
| C heifers | N | N | N | N | N | N | N | N |
| C calves | N | N | N | N | N | N | N | N |
| D lacting cow | N | N | N | N | N | N | P | N |
| D dry cow | N | N | N | N | N | N | N | N |
| D heifers | N | N | N | N | N | N | N | N |
| D calves | N | P | P | N | N | P | P | N |
| E lacting cow | N | N | N | N | N | N | N | N |
| E dry cow | N | N | N | N | N | N | N | N |
| E heifers | N | N | N | N | N | N | N | N |
| E calves | N | P | N | N | N | N | P | N |
| F lacting cow | N | N | N | N | N | P | N | N |
| F dry cow | N | N | N | N | N | P | N | N |
| F heifers | N | N | N | N | N | N | N | N |
| F calves | N | N | P | N | N | P | P | N |
| G lacting cow | N | N | N | N | N | P | N | N |
| G dry cow | N | N | N | N | N | P | N | N |
| G heifers | N | N | N | N | N | N | N | N |
| G calves | N | P | P | N | N | P | P | N |
| H lacting cow | N | N | N | N | N | N | N | N |
| H dry cow | N | N | N | N | N | N | N | N |
| H heifers | N | N | N | N | N | N | N | N |
| H calves | N | N | P | N | N | P | N | N |
| I lacting cow | N | N | N | N | N | N | N | N |
| I dry cow | N | N | N | N | N | N | N | N |
| I heifers | N | N | N | N | N | N | N | N |
| I calves | N | N | P | N | N | P | N | N |
| J lacting cow | N | N | N | N | N | N | N | N |
| J dry cow | N | N | N | N | N | N | N | N |
| J heifers | N | N | N | N | N | N | N | N |
| J calves | N | N | P | N | N | P | N | N |

P= positive, N= negative

**Supplementary Table 3.** Relative abundances of ARGs. The abundance of ARGs in the collected samples was quantified by ddPCR and expressed as gene copies/16S rRNA gene copy.

| Farms and categories | *bla*_TEM_ | *erm*B | *sul*2 | *tet*A |
| --- | --- | --- | --- | --- |
| A lacting cow | 9.04E-05 | 1.92E-04 | 3.56E-05 | 0.00E+00 |
| A dry cow | 1.89E-04 | 1.06E-03 | 5.43E-05 | 1.09E-04 |
| A heifers | 1.20E-03 | 9.40E-04 | 0.00E+00 | 1.66E-04 |
| A calves | 2.97E-03 | 1.94E-01 | 1.82E-01 | 7.50E-03 |
| B lacting cow | 3.76E-04 | 1.29E-03 | 3.64E-05 | 0.00E+00 |
| B dry cow | 1.36E-03 | 1.60E-03 | 1.29E-03 | 0.00E+00 |
| B heifers | 1.74E-03 | 5.14E-04 | 0.00E+00 | 0.00E+00 |
| B calves | 4.60E-04 | 3.36E-02 | 2.14E-03 | 1.76E-04 |
| C lacting cow | 1.03E-04 | 1.29E-03 | 6.50E-06 | 3.37E-05 |
| C dry cow | 1.11E-03 | 2.22E-04 | 3.95E-04 | 0.00E+00 |
| C heifers | 8.06E-04 | 1.76E-04 | 2.37E-03 | 0.00E+00 |
| C calves | 5.31E-04 | 4.54E-03 | 9.21E-03 | 1.93E-04 |
| D lacting cow | 7.56E-04 | 1.29E-03 | 4.70E-03 | 1.98E-03 |
| D dry cow | 1.19E-03 | 1.46E-03 | 1.03E-02 | 0.00E+00 |
| D heifers | 9.70E-04 | 9.19E-04 | 1.54E-02 | 0.00E+00 |
| D calves | 1.40E-02 | 2.96E-01 | 1.35E-03 | 9.52E-03 |
| E lacting cow | 5.80E-04 | 1.37E-03 | 2.86E-04 | 0.00E+00 |
| E dry cow | 8.92E-04 | 2.71E-03 | 3.26E-03 | 0.00E+00 |
| E heifers | 5.36E-04 | 3.50E-04 | 0.00E+00 | 0.00E+00 |
| E calves | 5.37E-02 | 8.33E-03 | 8.80E-03 | 3.89E-02 |
| F lacting cow | 5.26E-05 | 1.27E-04 | 4.05E-03 | 1.81E-05 |
| F dry cow | 2.67E-04 | 9.73E-05 | 6.15E-03 | 9.57E-05 |
| F heifers | 1.46E-04 | 3.38E-05 | 5.13E-03 | 1.74E-05 |
| F calves | 2.44E-03 | 3.05E-02 | 6.24E-02 | 5.30E-03 |
| G lacting cow | 1.94E-04 | 1.38E-04 | 8.00E-03 | 4.38E-05 |
| G dry cow | 1.71E-04 | 2.94E-05 | 9.65E-03 | 1.28E-05 |
| G heifers | 8.97E-05 | 6.41E-05 | 9.62E-03 | 5.31E-05 |
| G calves | 5.14E-03 | 1.81E-02 | 1.62E-02 | 5.51E-03 |
| H lacting cow | 4.59E-05 | 1.62E-04 | 7.67E-03 | 4.59E-05 |
| H dry cow | 3.47E-04 | 4.73E-04 | 1.44E-02 | 2.21E-05 |
| H heifers | 9.00E-05 | 2.93E-04 | 1.57E-03 | 0.00E+00 |
| H calves | 5.00E-04 | 3.19E-02 | 5.02E-02 | 1.16E-03 |
| I lacting cow | 3.62E-05 | 6.58E-04 | 6.04E-04 | 9.40E-06 |
| I dry cow | 1.41E-04 | 1.16E-04 | 4.86E-03 | 1.98E-05 |
| I heifers | 9.65E-04 | 1.40E-04 | 1.09E-02 | 0.00E+00 |
| I calves | 6.64E-04 | 6.60E-02 | 1.64E-02 | 7.13E-04 |
| J lacting cow | 9.66E-04 | 3.59E-03 | 2.96E-02 | 0.00E+00 |
| J dry cow | 8.24E-04 | 3.64E-04 | 1.03E-02 | 0.00E+00 |
| J heifers | 3.51E-04 | 1.82E-03 | 9.78E-03 | 0.00E+00 |
| J calves | 2.51E-02 | 2.50E-02 | 6.99E-02 | 1.68E-03 |

**Supplementary Table 4.** Statistical results for the multivariate analysis of variance (MANOVA) assessing the influence of the experimental variables (animal category and farm) on the general trend of the abundances of tested genes.

|  | **Df** | **Pillai** | **approx. F** | **num. Df** | **den. Df** | **p-value** |
| --- | --- | --- | --- | --- | --- | --- |
| Category | 2 | 0. 8754 | 2.6782 | 12 | 78 | 0.004572 ** |
| Farm | 9 | 1.3116 | 1.4637 | 36 | 108 | 0.069202 |
